# The thermal niche of Neotropical nectar‐feeding bats: Its evolution and application to predict responses to global warming

**DOI:** 10.1002/ece3.3171

**Published:** 2017-07-21

**Authors:** Stephanie Ortega‐García, Lázaro Guevara, Joaquín Arroyo‐Cabrales, Roberto Lindig‐Cisneros, Enrique Martínez‐Meyer, Ernesto Vega, Jorge E. Schondube

**Affiliations:** ^1^ Posgrado en Ciencias Biológicas Universidad Nacional Autónoma de México Coyoacán, Ciudad de México México; ^2^ Instituto de Investigaciones en Ecosistemas y Sustentabilidad Universidad Nacional Autónoma de México Morelia Michoacán México; ^3^ Departamento de Biología Evolutiva Facultad de Ciencias Universidad Nacional Autónoma de México Ciudad de México México; ^4^ Instituto Nacional de Antropología e Historia Ciudad de México México; ^5^ Instituto de Biología Departamento de Zoología Universidad Nacional Autónoma de México Coyoacán, Ciudad de México México

**Keywords:** Glossophaginae, physiology, records, resistance, temperature, tolerance

## Abstract

The thermal niche of a species is one of the main determinants of its ecology and biogeography. In this study, we determined the thermal niche of 23 species of Neotropical nectar‐feeding bats of the subfamily Glossophaginae (Chiroptera, Phyllostomidae). We calculated their thermal niches using temperature data obtained from collection records, by generating a distribution curve of the maximum and minimum temperatures per locality, and using the inflection points of the temperature distributions to estimate the species optimal (STZ) and suboptimal (SRZ) zones of the thermal niche. Additionally, by mapping the values of the STZ and SRZ on a phylogeny of the group, we generated a hypothesis of the evolution of the thermal niches of this clade of nectar‐feeding bats. Finally, we used the characteristics of their thermal niches to predict the responses of these organisms to climate change. We found a large variation in the width and limits of the thermal niches of nectar‐feeding bats. Additionally, while the upper limits of the thermal niches varied little among species, their lower limits differ wildly. The ancestral reconstruction of the thermal niche indicated that this group of Neotropical bats evolved under cooler temperatures. The two clades inside the Glossophaginae differ in the evolution of their thermal niches, with most members of the clade Choeronycterines evolving “colder” thermal niches, while the majority of the species in the clade Glossophagines evolving “warmer” thermal niches. By comparing thermal niches with climate change models, we found that all species could be affected by an increase of 1°C in temperature at the end of this century. This suggests that even nocturnal species could suffer important physiological costs from global warming. Our study highlights the value of scientific collections to obtain ecologically significant physiological data for a large number of species.

## 1 INTRODUCTION

The thermal niche of a species, defined as the range of temperatures where it is able to live, is one of the main determinants of its ecology and biogeography (Bozinovic, Ferri‐Yáñez, Naya, Araújo, & Naya, 2014). It is determined by the species size and shape, and its ability to survive in, or adapt to places with different temperature regimes (Angilletta, Niewiarowski, & Navas, 2002; Porter & Kearney, 2009). It is strongly associated with the species metabolic costs and its physiological capacities to withstand thermal variation (Spicer & Gaston, 1999). From an energetic perspective, it is closely limited by the species capacity to provide the energy needed to maintain its metabolic costs under different ambient temperatures (Bell, Bartholomew, & Nagy, 1986).

Endothermy is a physiological strategy mainly used by mammals and birds, which allow them to maintain an almost constant body temperature, independently from ambient temperature (Schmidt‐Nielsen, 1997). It is achieved by using the heat generated by body functions to control body temperature within a range of environmental conditions that favors its metabolic functions, enabling it to survive in places with highly variable conditions (Angilletta et al., 2002). Overall, this internal heat is the incidental result of the routine metabolism of animals, and its costs are energetically low (Bozinovic et al., 2014). However, when endothermic animals are confronted with extreme temperatures, they use specialized mechanisms to maintain stable body temperature (i.e., the use of large amounts of energy to increase heat production, or water loss by evapotranspiration to cool down; Scholander, Hock, Walters, Johnson, & Irving, 1950). As a result of this, by facing different ambient temperatures along its geographic distribution, a species confronts areas within its thermal niche that have different metabolic costs, some of them low, but some exceptionally high (Angilletta et al., 2002).

The division of the thermal niche of a species in areas of optimal conditions, with low physiological costs, and areas of suboptimal conditions, with higher physiological costs, is not a new idea in the field of ecological physiology. Brett (1952) defined the area where environmental conditions are optimal for the survival of the members of a species as its tolerance zone (TZ), and the zone where environmental conditions reduce individual survival by increasing physiological costs, as the species resistance zone (RZ). Regarding the thermal niche and physiological capabilities of endotherm animals, the TZ corresponds to temperature ranges where species have lower metabolic rates or present metabolic costs, that while higher, can be easily covered by their energy intake, reducing their effects on the animal capacity to survive and reproduce. Some authors have suggested that this temperature range is associated with the species thermoneutral zone, because the thermoneutral zone limits provide an index of an endotherm's temperature comfort range (Bozinovic et al., 2014; McNab, 2012). The RZ comprises temperatures above and below the tolerance zone, where metabolic costs increase as the species move away from the thermoneutral zone to a point where individuals cannot survive for long periods of time (Cossins & Bowler, 1987).

Despite the fact that understanding the TZ and the RZ of different species, and its evolution, allow us to understand the thermal ecology and capacity of animals to adapt and survive climate change, we have scarce knowledge of the thermal tolerances of most organisms (Araújo et al., 2013). This is mainly the result of the demanding experimental methods needed to determine the thermal limits of animals. Additionally, laboratory measurements of metabolic responses of animals to different temperatures (e.g., thermoneutral zones) cannot be used to infer the capacity of animals to find their thermal niches in complex natural environments (Porter & Kearney, 2009). However, with the inclusion of geographic information system (GIS) in scientific disciplines like biology, we can infer a species’ TZ and RZ using temperature information related to geographic information data that can be found in museum specimens. This geographically linked information offers us the advantage of integrating the interaction between physiological capacities and environmental factors over large geographic areas, allowing us to obtain physiological information at the level of species, and not only at level of individuals. Furthermore, this information combined with phylogenetic approaches has the potential to provide insights on the evolution of the thermal niches of animals.

The family Phyllostomidae comprises a clade of bats endemic to the Americas (Fleming, Geiselman, & Kress, 2009). This group presents the biggest diversity of diets for a family of vertebrates (Gardner, 1977), including specialized nectarivory, a diet found in the members of the subfamily Glossophaginae. The species of this diverse clade display several features associated with their sweet diet, such as long and narrow snouts, a reduction in the number of functional teeth, an elongated and projectable tongue, and several digestive and renal traits that allow them to cope with their sugary water diet (Carstens, Lundrigan, & Myers, 2002; Schondube, Herrera‐M, & Martínez del Rio, 2001). As a result of their dependence on floral nectar as a source of energy, the evolution of this group of nectar‐feeding bats occurred in the tropics, where the diversity of plants is high, forming close‐ties with the plants species from which they obtain their food (von Helversen & Winter, 2003; Valiente‐Banuet, Arizmendi, Rojas‐Martínez, & Domínguez‐Canseco, 1996). Because these mammals have high metabolic rates (Voigt & Speakman, 2007), and nectar is a resource that varies widely in time and space (Chalcoff, Aizen, & Galetto, 2006), they tend to live on the verge of a negative energy balance (Ayala‐Berdon, Schondube, Stoner, Rodriguez‐Peña, & Martínez Del Rio, 2008; Ayala‐Berdon et al., 2011; Cruz‐Neto & Abe, 1997; von Helversen & Winter, 2003). Consequently, we can expect nectar‐feeding bats to be very sensitive to extreme temperatures, and the metabolic costs associated with them.

In this study, we determined the thermal niche of 23 species of nectar‐feeding Neotropical bats based on collection records from public databases. Additionally, by using a phylogenetic approach, we propose a hypothesis of the evolution of the thermal niche in Glossophaginae. Finally, we related the characteristics of the thermal niches with some of the physiological capacities of these bats, and the ecological conditions in which these species have evolved. We highlight the usefulness of public databases, along with spatial tools, to reveal critical insights to understand the evolution of the thermal niches of bats and their potential adaptation capabilities at a time in which planetary climate is changing fast due to anthropogenic factors (Parmesan & Yohe, 2003).

## 2 MATERIALS AND METHODS

### 2.1 Taxon sampling

We included in our study 23 extant species belonging to the subfamily Glossophaginae (Rojas, Warsi, & Dávalos, 2016). This subfamily is subdivided in two clades (following Carstens et al., 2002): “Glossophagines” (*Glossophaga commissarisi, G. leachii, G. longirostris, G. morenoi, G. soricina, Leptonycteris curasoae, L. nivalis, L. yerbabuenae, Monophyllus plethodon*,* M. redmani, Phyllonycteris poeyi, Erophylla sezekorni, and Brachyphylla nana*), and “Choeronycterines” (*Anoura geoffroyi, A. caudifer, A. cultrata, A. latidens, Choeroniscus godmani, C. minor, Choeronycteris mexicana, Hylonycteris underwoodi, Lichonycteris obscura* and *Musonycteris harrisoni*; Carstens et al., 2002; Simmons, 2005; Rojas et al., 2016).

Locality records for each species were obtained from the Global Biodiversity Information Facility (GBIF; www.gbif.org). We treated databases conservatively by comparing each locality collection with the areas of species distribution based on Hall (1981), Gardner (2007), and Reid (2009). All collection records that were clearly outside the mentioned ranges were excluded from further analyses (following Elith et al., 2006 and Jaramillo & Martínez, 2014). All records with the same geographic coordinates were considered as a single locality. As small differences in geographic coordinates might represent important differences in environmental conditions, especially when a geographic region presents a complex topography (i.e., mountain regions of Mexico, Central America, and the Andes), nearby collection points (≥1 km) were considered as independent localities. The number of collection localities for each species is shown in Table 1. Additional data on all the unique localities used for our analysis can be consulted in Appendix S1 (supporting information).

**Table 1:** Species Tolerance Zone (STZ) and Species Resistance Zone (SRZ) values for the 23 bat species and the outgroup (^a^) included in our study. *n *= records number

Species	*n*	SRZ_lower_	STZ_lower_ ‐STZ_upper_	SRZ_upper_
*Pteronotus parnellii* ^a^	1273	0.7	18.8–28.6	35
*Glossophaga longirostris* ^b^	202	12.2	21.9–30.7	32.7
*Glossophaga leachii*	270	4.8	15.3–30	34.2
*Glossophaga morenoi*	175	4.5	16.5–29.3	34.6
*Glossophaga commissarisi*	401	5.8	16.9–29.2	34.2
*Glossophaga soricina*	2 404	0.6	17.5–28.6	35
*Leptonycteris curasoae* ^b^	20	16.6	22.4–30.4	32.6
*Leptonycteris nivalis* ^b^	129	0.9	8.5–24.6	33.8
*Leptonycteris yerbabuenae*	403	0	11.5–27.3	34.6
*Monophyllus plethodon*	52	12.8	19.2–27.1	28.9
*Monophyllus redmani*	113	2.6	18.3–28.7	30.4
*Phyllonycteris poeyi*	25	9.8	17.5–29.7	29.8
*Erophylla sezekorni* ^b^	87	7.8	17.7–28.1	31
*Brachyphylla nana* ^b^	36	9.8	17.2–27.8	30
*Musonycteris harrisoni* ^b^	20	11.4	16–31.4	33.6
*Choeronycteris mexicana*	285	0	6.3–23.5	32.2
*Choreoniscus godmani*	96	5.3	19.3–30.1	32.9
*Choreoniscus minor* ^b^	41	8.5	20.6–30	31.2
*Hylonycteris underwoodi* ^b^	98	0	13.7–25.8	32.4
*Lichonycteris obscura* ^b^	28	11.9	21.3–28.7	32
*Anoura latidens* ^b^	23	7.9	20.4–29.2	31.4
*Anoura geoffroyi*	691	0	10.7–24.6	33.6
*Anoura caudifer*	217	0.4	15.1–26.5	32.6
*Anoura cultrata*	43	0	16.5–27.7	30.1

^b^Represent species that did not showed stabilized variances in temperature data.

### 2.2 Calculation of the thermal niches

Our concept of thermal niche includes an important difference in the definition of TZ and RZ from the concept generated by the work of Brett (1952). While TZ and RZ were defined in the past at an individual level, measuring the survival time of individual animals at different temperatures, our concept reflects the response of a species to environmental temperatures. We determined the TZ and RZ of Glossophaginae bats using our unique localities database. For each of our studied bat species, we plotted the distribution of the number of localities with different temperatures and used the inflection point of the distribution curve to determine the limits between the TZ and the RZ. To avoid having a confusion with the previous definitions of TZ and RZ, we renamed the elements of the thermal niche as Species Tolerance Zone (STZ) and Species Resistance Zone (SRZ).

Our use of the inflection points as representatives of the limits of the thermal STZ and SRZ is based on the following assumption: because individuals should have higher survival rates in the STZ, the number of localities where the species has been collected should be larger for the range of temperatures that are found inside the STZ than for the SRZ. As a result of this, our concept of STZ includes the temperatures that allow the species to be abundant and reflect the conditions the species had adapted throughout its evolution, while the SRZ comprises temperatures in which only some populations of the species can survive and represent extreme conditions where the species is evolving to adjust to new conditions (areas of strong selection sensu Sexton, McIntyre, Angert, & Rice, 2009).

To describe the thermal niche, we generated a database of minimum and maximum temperatures of all unique localities for each species. We used monthly temperature data obtained at www.worldclim.org (Hijmans, Cameron, Parra, Jones, & Jarvis, 2005). This procedure was performed using ESRI ArcGIS © version 10 (Redlands, CA 1999–2010). For minimum temperatures, we used the value of the coldest temperature recorded for each locality. Because bats are nocturnal animals that use shelters during the day, they are not exposed to daily maximum temperatures. Unfortunately, there is no available information on maximum night temperatures at global level. Therefore, to determine the maximum temperature limits of the thermal niche of bats, we used the minimum value of the maximum temperatures recorded at each locality as a conservative proxy for maximum temperatures that bats could find at night.

**Figure 1:**
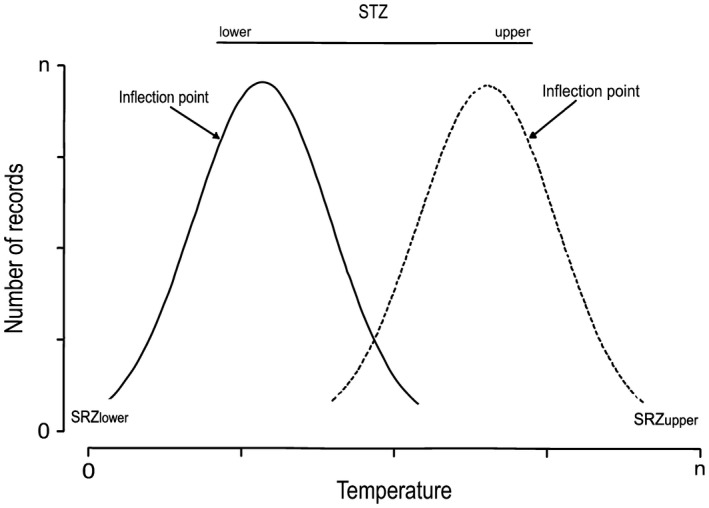
Method used to determine the thermal niche of Neotropical nectar‐feeding bats. We delimitated the Species Tolerance Zone (STZ) and Species Resistance Zone (SRZ) using a database of unique localities. We constructed two curves for each species, one for the minimum temperatures (solid line), and the second for the maximum temperatures (dash line). We calculated the left inflection point for the minimum temperature curve (STZ_lower_), and the right inflection point for the maximum temperature curve (STZ_upper_), while the limits of SRZ were calculated using the minimum (SRZ_lower_) and maximum values (RSZ_upper_) of each one of the temperature distribution curves. See methods section for more details

We then calculated the thermal niche obtaining the STZ and SRZ separately for each species. As previously mentioned, to calculate thermal STZ and SRZ, we constructed two curves for each species, one for the minimum temperatures, and the second for the maximum temperatures. We calculated the left inflection point for the minimum temperature curve (STZ_lower_), and the right inflection point for the maximum temperature curve (STZ_upper_; Figure 1). We considered the two inflection points as the limits of the STZ, while the limits of SRZ were calculated using the minimum (SRZ_lower_) and maximum values (SRZ_upper_) of each one of the temperature distribution curves (Figure 1). Inflection points were calculated using the R package Inflection V.1.1 (Christopoulos, 2013; R Development Core Team 2016). Additionally, we determined the thermal niche breadth of each species by calculating the total number of °C that exist between the upper and lower limits of the SRZ.

Because the total number of unique localities was highly variable among species of bats, we explored the effect of increasing sample size on variance of the STZ and SRZ values. In particular, we were interested in evaluating whether the variance became stable for the given sample size of each species. To do this, we implemented a script with R (R Core Team, 2016) to plot the variance as the sample size increased from one to *n*, where *n* is the sample size for each species (Appendix S2). The plot allowed us to conduct a visual exploration of the tendency of the variance as sample size increased.

### 2.3 Reconstruction of ancestral states and evolution of thermal niche in nectar‐feeding bats

Understanding the evolution of thermal niches requires a robustly supported phylogenetic hypothesis as framework. Thus, our taxon sampling is in line with the current knowledge of the evolutionary history of nectar‐feeding bats (Amador, Moyers Arévalo, Almeida, Catalano, & Giannini, 2016; Carstens et al., 2002; Dávalos, Velazco, Warsi, Smits, & Simmons, 2014; Rojas et al., 2016). Despite historical debates about possible phylogenetic relationships within the family Phyllostomidae (Dávalos et al., 2014), we follow a recent proposal for the subfamily Glossophaginae based on a multi‐locus analysis (Rojas et al., 2016). Phylogenetic hypothesis within the subfamily Glossophaginae are well‐supported and, in general, they agree with the current understanding of the systematics of the group (*e.g.,* Amador et al., 2016; Dávalos et al., 2014). We sampled 100% of the genera and 88% of the species of the subfamily Glossophaginae analyzed by Rojas et al. (2016). In addition to the species considered by Rojas et al. (2016), we included *Leptonycteris nivalis* in our analyses.

In order to investigate how STZ and SRZ have changed throughout the evolutionary history of nectar‐feeding bats, we inferred the ancestral states of all four elements of their thermal niche (STZ_lower_, STZ_upper_, SRZ_lower_, and SRZ_upper_). As mentioned previously, in order to conduct the reconstruction of ancestral states and the evolution of the thermal niche of nectar‐feeding bats, we followed the phylogenetic hypothesis from Rojas et al. (2016). To place *L. nivalis* in that phylogenetic hypothesis, we ran a Bayesian inference using the 119 morphological characters described by Carstens et al. (2002), and 658 base pairs of the mitochondrial gen Cytochrome Oxidase subunit 1. Even though our phylogenetic analyses included only a subset of the full sampling, and different characters from those used in Rojas et al. (2016), it was adequate to determine the position of *L. nivalis* relative to other congeneric species. These analyses kept morphology and genetic data separately and were based on eight independent runs and 10,000,000 generations. We used the Common Mustached Bat, *Pteronotus parnellii*, as outgroup (Mormoopidae). Bayesian analysis was performed in MrBayes version 3.2 (Huelsenbeck, Ronquist, Nielsen, & Bolback, 2001; Ronquist et al., 2012).

Because our focus is the ancestral reconstruction of the thermal niche using a well‐supported phylogeny, we replicated a tree in Newick format containing the phylogenetic relationships for the Glossophaginae from Rojas et al. (2016) plus the addition of *L. nivalis*. The reconstruction of ancestral states was conducted on this tree using the method of parsimony for the STZ_lower_, STZ_upper_, SRZ_lower_ and SRZ_upper_ data using Mesquite V.3.04 (Maddison & Maddison, 2015). In a descriptive manner, we used “+” and “−” symbols to indicate species that presented a higher or lower temperature value in relation to their ancestral state in figure 3. In similar fashion, we used “0” if we did not found changes between the ancestral value of the thermal variable and the values calculated for the current thermal niches.

Finally, we conducted phylogenetic signal analyses on the TZ_lower_, STZ_upper_, SRZ_lower_ and SRZ_upper_ data of the bat species included in our phylogeny. Phylogenetic signal is defined “as a tendency for related species to resemble each other more than they resemble species drawn at random from the tree” (Blomberg & Garland, 2002). To determine the existence of a phylogenetic signal, we calculated Blomberg's *K* (Blomberg, Garland, & Ives, 2003) using R package Picante (v. 3.0.2) (Kembel et al., 2010; R Core Team, 2016).

## 3 RESULTS

### 3.1 Effect of sample size on thermal niche data

Change in variance in ambient temperature values in response to sample size differed considerably among species. As expected, for species collected in a small number of localities, such as *Choeroniscus minor, Lichonycteris obscura, Musonycteris harrisoni, Anoura latidens, Leptonycteris curasoae*,* Brachypylla nana* and *Erophylla sezekorni*, variance did not stabilize as sample size increased. But this lack of stabilization also occurred for maximum temperatures for three species with larger data sets: *Leptonycteris nivalis* (129 records), *Glossophaga longirostris* (199) and *Hylonycteris underwoodi* (98). However, this did not occur for minimum temperatures. Both for maximum and minimum temperatures, sample sizes for detecting variance stabilization varied widely, from 25 records in *Monophyllus plethodon,* to five hundred in *Glossophaga soricina*. Overall, for all the 23 species included in this study, sixteen species had variances that stabilized for minimum temperatures, and thirteen species showed stabilized variances for maximum temperatures. We decided to include species that did not showed stabilized variances in temperature data in our study because there is very little information on those species (see Table 1), and our analyses provide a starting point to understand their thermal biology, and to promote research on this topic. For these species, our results need to be considered as preliminary.

### 3.2 The thermal niche of Neotropical nectar‐feeding bats

**Figure 2:**
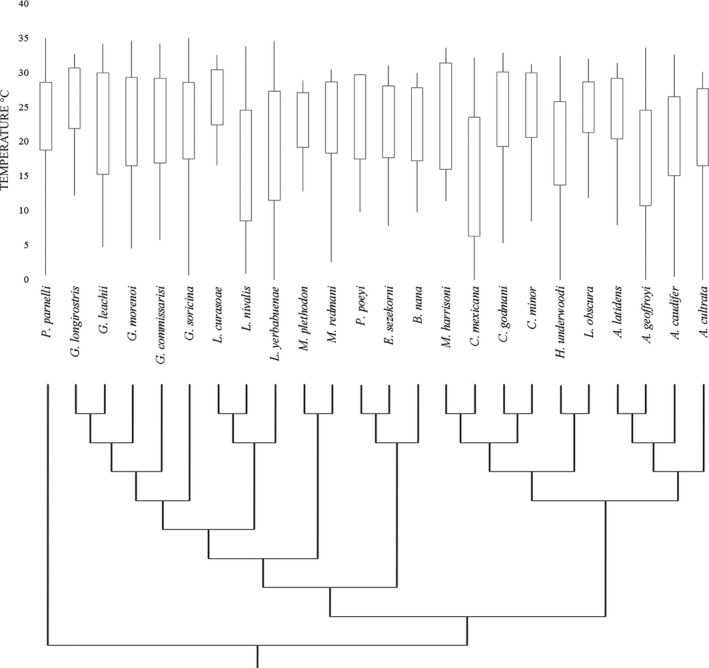
Thermal niches of Neotropical nectar‐feeding bats in relation to their phylogeny. The phylogeny was modified from Rojas et al. (2016). The STZ is represented by the boxes, while the SRZ is represented by the whiskers, temperature is in °C. To understand the ancestral states of the thermal niche of the members of this clade, we included one species as an out group to the family Phyllostomidae (*Pteronotus parnellii*) in our analyses

Nectar‐feeding bats thermal niches showed a high variability in the SRZ_lower_ limit (5.5 ± 5.3°C, mean ± *SD*), while the SRZ_upper_ limit tended to be similar among species (32.5 ± 1.6°C), and did not exceed 35°C for any species (Table 1, Figure 2), even though higher temperatures were present in the geographic distribution of our study species (e.g., 36°C). A similar pattern exists for the temperature values of the STZ, with the STZ_lower_ limit values showing a higher variability (16.4 ± 4.3°C) than the STZ_upper_ limit values (28.1 ± 2.1°C; Table 1, Figure 2). The total breadth of the thermal niche varied widely among species. The species with the smallest thermal niche was *Leptonycteris curasoae* with a range of 16°C*,* while the larger thermal niche was found in *L. yerbabuenae* (34.6°C). Of the 23 species in our study, only five (*L. nivalis, L. yerbabuenae, Hylonycteris underwoodi, Choeronycteris mexicana* and *Anoura geoffroyi*) extended their tolerance zone limits below 15°C. Thermal niches values for the 23 species can be found in Table 1.

### 3.3 Ancestral states of the thermal niche

**Figure 3:**
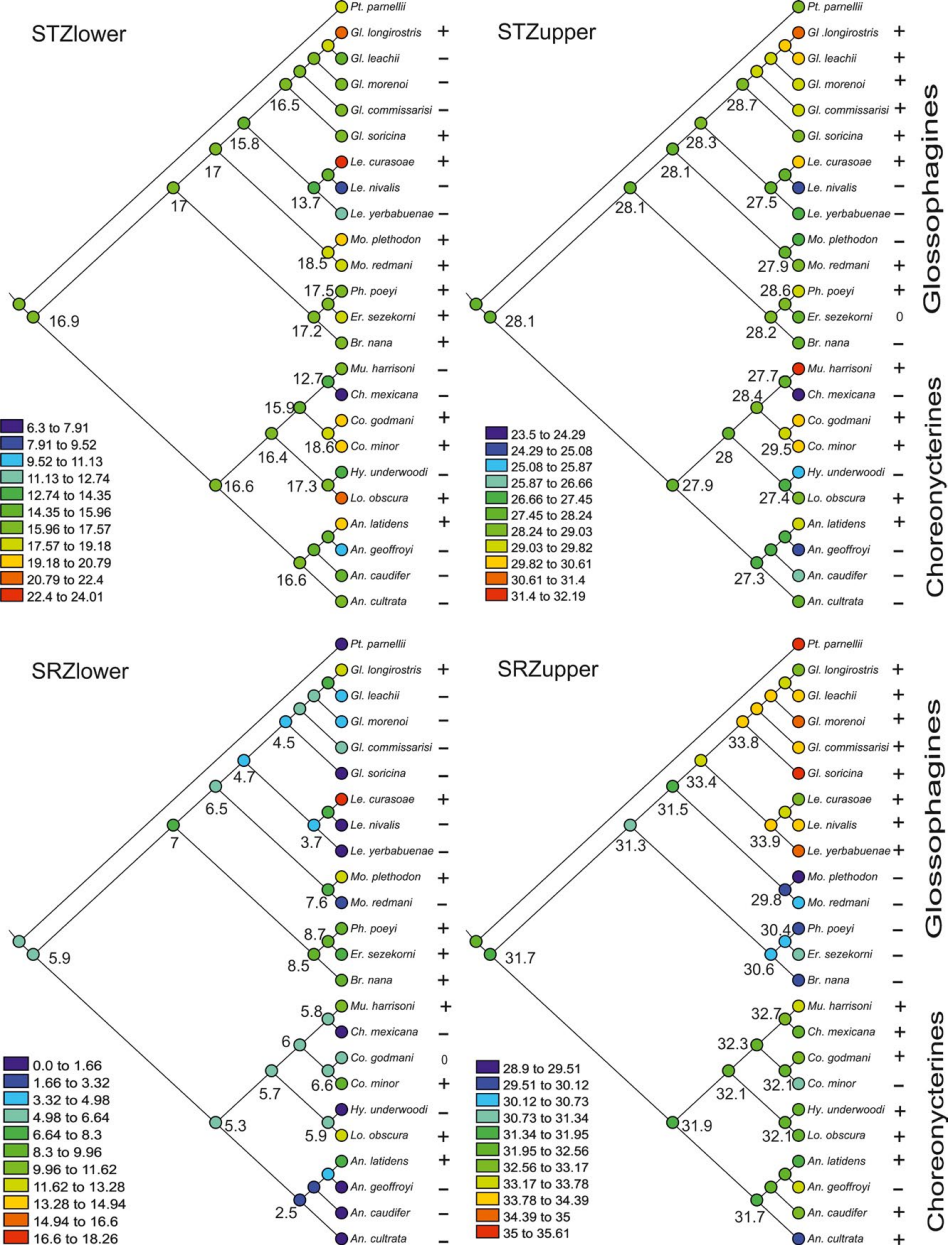
Reconstruction of the ancestral states of the four elements of the thermal niche of Neotropical nectar‐feeding bats. The temperature scale (in °C) differs in each one of the panels. Positive signs (+) represent an increment in the temperature value of a species in relation to the common ancestor of the subfamily Glossophaginae, while negative signs (−) represent a decrease in temperature, and zeros (0) a lack of change

The values of the ancestral state of the four elements of the thermal niche for the subfamily Glossophaginae had lower values than those present in the thermal niches of some of the extant species included in our analyses. Values of the ancestral state for the thermal niche elements of nectar‐feeding bats in this clade were: RZ_lower_ = 5.9°C, TZ_lower_ = 16.9°C, TZ_upper_ = 28.1°C and RZ_upper_ = 31.7°C (Figure 3).

When we compared the ancestral state of the thermal niche of the Glossophagines and Choeronycterines, we found that the values of three of the four elements of the thermal niche for the clade Choeronycterines had lower temperature values (SRZ_lower_ = 5.3°C, STZ_lower_ = 16.6°C, and STZ_upper_ = 27.9°C), than those expressed by the ancestor of the clade Glossophagines (SRZ_lower_ = 7°C, STZ_lower_ = 17°C, and STZ_upper_ = 28.1°C). Additionally, the ancestral values for these three elements of the thermal niche of the Choeronycterines had lower values than the ancestral state of the whole subfamily. However, the ancestral state value of SRZ_upper_ in Choeronycterines (SRZ_upper_ = 31.9°C) was higher than the value for Glossophagines (SRZ_upper_ = 31.3°C). When we looked at the values of the ancestral states of three of the four elements of the thermal niche (STZ_lower_, STZ_upper_ and SRZ_lower_) closer to the tips of the branches, we found that three clades had temperature values lower than the ancestor of the subfamily Glossophaginae: 1) the ancestor of the genus *Anoura*, 2) the ancestor of the clade containing *Choeronycteris* and *Musonycteris*, and 3) the ancestor of the genus *Leptonycteris*; Figure 3). The ancestral state values of SRZ_upper_ closer to tip of the branches had values that did not differ with the ancestor of the subfamily (ancestor of the genus *Anoura*), or were a litter higher (ancestor of the clade containing *Choeronycteris* and *Musonycteris,* and the ancestor of the genus *Leptonycteris*; Figure 3).

Our phylogenetic signal analyses indicate that SRZ_upper_ presented a phylogenetic signal (*K* = 0.95, *p* < .001). However, the other three elements of the thermal niche did not (STZ_lower_: *K* = 0.23, *p* = .95; STZ_upper_: *K* = 0.24, *p* = .95; and SRZ_lower_: *K* = 0.24, *p* = .97).

## 4 DISCUSSION

Thermal niches of the nectar‐feeding bats of the subfamily Glossophaginae had a low variation in their upper temperature values, but show a high variation in the limits of their tolerance and resistance zones for lower temperatures. Additionally, the values of the ancestral state for the different components of their thermal niche were associated with temperatures in the lower end of the spectrum showed by most of the extant species in the group, suggesting that this group evolved under colder weather conditions. In this section, we first compare the results from our novel method to calculate thermal niches with those of previously published studies on the thermal biology of bats. Second, we explore the existence of niche conservatism in the upper limits of the thermal Species Resistance Zone. Third, we discuss the evolution of the thermal niche in this clade of nectar‐feeding bats. And finally, we use the characteristics of the thermal niche to understand the capabilities of these organisms to withstand changes in ambient temperature generated by anthropogenic climate change.

### 4.1 Laboratory studies of thermal biology of nectar‐feeding bats

There is limited information on the metabolic responses to temperature of Glossophaginae bats from laboratory studies. The existing research has determined lethal temperatures, and/or metabolic curves (Scholander curves) for only seven species, and we do not have values of critical and lethal temperatures for all of them (Arends, Bonaccorso, & Genoud, 1995; Carpenter & Graham, 1967; Cruz‐Neto & Abe, 1997; McManus, 1975; McNab, 1969; Soriano, Ruiz, & Arends, 2002). The trait best studied in the laboratory is the lower critical temperature, for which we have data for only six species (A*noura latidens, Choeroniscus godmani, Glossophaga soricina, G. longirostris, Leptonycteris yerbabuenae*, and *L. curasoae*). Unexpectedly, the values of this trait did not correlate with the lower temperature values present in the geographic distributions of the species (*r* = −.36, *p* = .48). This lack of correlation limits our capacity to use laboratory information to understand the thermal ecology of this group of bats in the field.

While laboratory studies provide an important base to understand the thermic physiology of endothermic animals, and the results for some taxa correlate with temperature values measured in the field at the sites where the different species live (see Bozinovic et al., 2014), they represent a “limited” view of the fundamental thermal niche of animals. Laboratory studies, by measuring the metabolic responses of endotherms to ambient temperatures under controlled, and therefore unnatural conditions (i.e., fasting animals with limited movement during short periods of time; McNab, 1989; Arends et al., 1995), ignore the capacity of animals to obtain energy, or use energy reserves, and do not allow us to determine the net energetic cost of a shift in ambient temperature for the bats. If an animal has a high capacity to acquire energy, like nectar‐feeding bats do, a small, or even a large, increase in metabolic costs due to thermoregulation could be irrelevant for the species under natural conditions but not in the laboratory when experimental individuals were fasting (Ayala‐Berdon, Schondube, & Stoner, 2009). This could explain why the lower critical temperature data was not correlated with minimum field temperatures. This problem may seriously cripple our capability to use metabolic curves to understand the thermal niches of nectar‐feeding bats in a real ecological context.

Geographic presence data from natural history museums records integrate the physiological characteristics of species (that define their fundamental niche) with environmental factors. The intersection of intrinsic (physiology) and extrinsic factors (bionomic and scenopoetic niche axes sensu Hutchinson′s 1978), determine the capacity of a species to be present and survive at a given geographic site (Peterson et al., 2011). While from a geographic locality we can obtain environmental (i.e., temperature, precipitation among others), and topographic data (i.e., elevation, slope), this type of information also conceals data on microhabitat, species interactions and diet quality, by proving a biogeographic context for the species (Peterson et al., 2011). This perspective of the niche, from the point of view of some of its axes, while myopic (sensu Newsome, Martinez del Rio, Bearhop, & Phillips, 2007), offers us a snapshot of the costs and benefits that animals face in the field, and provides critical information to understand when laboratory physiological data are relevant to understand the ecology of a species.

### 4.2 Upper and lower limits of the thermal niche in nectar‐feeding bats

Our results indicate that nectar‐feeding bats have an average SRZ_upper_ value of 32.5°C ± 1.6°C, while the mean value of the SRZ_lower_ was 5.5 ± 5.3°C. We also found that the SRZ_upper_ values showed a phylogenetic signal. The SRZ_upper_ values we found in our research are similar to those reported by a study that synthesized the thermal tolerances of a large number of terrestrial ectotherm and endotherm organisms from a wide arrange of geographic areas (Araújo et al., 2013), and those of a comparative study of 85 species of rodents (Bozinovic et al., 2014). Both Araújo et al. (2013) and Bozinovic et al. (2014) found that the upper limit of the thermal niche was shared by most species of mammals, and was located close to 34°C, while the lower limit of the thermal niche was labile. In our study group, this low variation in the thermal upper limits of the species can be explained by two complementary hypotheses: (1) high environmental temperatures are less variable than cold temperatures (Addo‐Bediako, Chown, & Gaston, 2000; Boher, Godoy‐Herrera, & Bozinovic, 2010), generating an upper limit to the thermal niche that varies less than its lower limit (Araújo et al., 2013; Bozinovic et al., 2014) and (2) the SRZ_upper_ values could be limited by negative effects of high temperature on cell membranes, and protein structure and function, while the SRZ_lower_ values would be limited by the capacity of the different species to obtain the energy needed to survive cold conditions. This would allow the higher limits of the thermal niche to be controlled by biochemical thermal limits, while the lower limits of the thermal niche could vary more in response to differences in energetic acquisition/thermodynamic effects of species present in colder localities (Araújo et al., 2013).

### 4.3 The evolution of thermal niches in nectar‐feeding phyllostomid bats

The Family Phyllostomidae originated between the Oligocene and the Early Miocene in the northern part of South America and/or the Antilles (29–20 MYA; Czaplewski, Takai, Naeher, Shigehara, & Setoguchi, 2003; Datzmann, von Helversen, & Mayer, 2010; Rojas et al., 2016), with the first nectar‐feeding species in the subfamily Glossophaginae appearing in the middle Miocene (25–17 MYA; Datzmann et al., 2010). During the Miocene, due to massive plate tectonics, substantial landscape and climate changes occurred in tropical America (Turchetto‐Zolet, Pinheiro, Salgueiro, & Palma‐Silva, 2013; Zachos, Schackleton, Revenaugh, Pälike, & Flower, 2001). Global climate cooled and was associated with an increase in aridity (Kürschner, Kvacek, & Dilcher, 2008; Zachos et al., 2001). Cool climate conditions present when this clade of nectar‐feeding bats diversified, support our results of a colder ancestral state of the thermal niche in this group of bats.

The two clades in the subfamily Glossophaginae showed differences in the values of the ancestral state of their thermal niche elements. The Choeronycterines (sensu Carstens et al., 2002), that include the genera *Anoura*,* Hylonycteris*,* Lichonycteris*,* Choeroniscus*,* Choeronycteris*,* Musonycteris*, and *Scleronycteris* (the latter not included in this study), had lower temperature values in their ancestral state of three of the four elements of their thermal niche (SRZ_lower_, STZ_lower_, STZ_upper_) and a similar value for SRZ_upper_ than the clade of the Glossophagines (genera *Glossophaga*,* Leptonycteris, Monophyllus, Phyllonycteris, Erophylla* and *Brachyphylla*). This suggests that the evolution of these two clades could have been the result of a divergent adaptation to different thermal conditions. The association of the Choeronycterines with colder temperatures is the result of most species in the genus *Anoura*, and other species such as *Hylonycteris underwoodi* and *Chorenycteris mexicana,* having colder thermal niches. Koopman (1981) proposed that the genus *Anoura* was “fairly primitive” (meaning basal) in this clade, suggesting that the Choeronycterines evolved associated with the cooler conditions found in the mountain areas of South and Central America, with a subsequent adaptation of some group to warmer conditions (i.e., genera *Choeroniscus*,* Lychonycteris*, and *Musonycteris*; Koopman, 1981; Gardner, 2007). This is supported by the altitudes at which these different genera of bats are generally found (Ceballos & Oliva, 2005; Eisenberg, 1989; Eisenberg & Redford, 1999; Gardner, 2007; Redford & Eisenberg, 1992).

Our results indicate that the clade of the Glossophagines had an ancestral state of their thermal lower limits related to warmer temperatures. This clade includes six genera associated with the humid and arid tropical low lands of South America, the Caribe, Central America and Mexico (Eisenberg, 1989; Gardner, 2007; Redford & Eisenberg, 1992; Rojas et al., 2016; Silva, 1979; Villa‐R., 1967). Our study suggests that this clade may have evolved their thermal niches as an adaptation to warmer climate conditions. Rojas et al. (2016) suggested that the basal clade of the Glossophagines (composed by the genera *Phyllonycteris, Erophylla* and *Brachyphylla*), evolved in the Antilles. So, the warmer thermal lower limits of the niches present in the basal members of the Glossophagines could be the origin of the changes of the thermal niche to warmer conditions in this clade, with only one species reverting this pattern, and gaining the capacity to invade the colder climate present in the mountain areas of Mexico and the southern part of the United States (*L. nivalis*; Arita, 2005).

### 4.4 Relationship between thermal niches and the ability of Neotropical nectar‐feeding bats to withstand global warming

By relating the thermal niches of our study species with models of climate change, we formulated a conservative hypothesis of the responses of nectar‐feeding bats to changes in ambient temperature. Several simulations of anthropogenic caused climate change (i.e., CMIP5, RCP4,5, RCP6,0, RCP8,5) project increases in temperature at the end of this century that vary from 1.5 to 2.8°C for different tropical and subtropical areas in America (Diffenbaugh & Giorgi, 2012; IPCC 2014). If we assume a conservative constant increase of 1°C across all localities, we observe that between 34.7% and 75% of the unique localities of the different species, shift their temperatures from inside the STZ to the SRZ, and between 0.4% and 28% of the localities move their temperatures from the SRZ to a value outside the SRZ_upper_ limit. This suggests that, even for species like bats, which are nocturnal, and do not confront extreme diurnal temperatures, global warming may pose an important direct threat. Additionally, our results show that the effect of global warming should vary widely among species. However, because we are assuming a constant temperature increase in all localities, the values of our projections need to be considered with caution.

Furthermore, we found a negative relationship between the percentage of localities that moved its temperature values outside the SRZ and the width of the thermal niche of the different species (*R* ^2^ = .19, *p* = .037). Additionally, the thermal niche width was positively related to the size of the geographic distribution area of our study species. The relationship between these two results suggests that species with restricted distributions could be more affected by global change, as have been previously stated by Walther et al. (2002).

Finally, our study shows that locality data obtained from natural history museums could provide crucial information to determine the physiological parameters of species. The method we proposed here to describe the thermal niche of Neotropical nectar‐feeding bats, by using temperature data linked to locality records, offers the possibility to work with large number of species, and generates physiological data that are ecologically relevant in a critical moment of history calling for urgent action to address anthropogenic climate change.

## CONFLICT OF INTEREST

None declared.
