# Supplementary material for: The thermal niche of Neotropical nectar‐feeding bats: Its evolution and application to predict responses to global warming
Source: Ecol Evol. 2017 Jul 21;7(17):6691–701. doi: 10.1002/ece3.3171 (PMC5587449; doi:10.1002/ece3.3171)
Supplement: Supplementary file 2 [file ECE3-7-6691-s002.docx]

Appendix S2. R script to analyse changes of variance in relation to sample size

The R script follows:

nums = scan(“Dataset”)

Dataset$V1

attach(Dataset)

sem<-numeric(n)

sem[1]<-NA

for(i in 2:n)sem[i]<-var(Dataset$V1[1:i])

plot(1:n,sem, main="*title*", xlab="*n*", ylab=“*variance*")

dev.copy(postscript, file="/home/roberto/Desktop/cluster.eps", height=6, width=6, horizontal=F, onefile=F)

dev.off()

In this script “Dataset” is replaced by the name of the file containing temperature data, and “n” is replaced with the number of data points.
